# Understanding non-communicable diseases: combining health surveillance with local knowledge to improve rural primary health care in South Africa

**DOI:** 10.1080/16549716.2020.1852781

**Published:** 2020-12-24

**Authors:** Eilidh Cowan, Lucia D’Ambruoso, Maria van der Merwe, Sophie Witter, Peter Byass, Soter Ameh, Ryan G. Wagner, Rhian Twine

**Affiliations:** a School of Geosciences, University of Edinburgh, Edinburgh, UK; b Aberdeen Centre for Health Data Science, Institute of Applied Health Sciences, University of Aberdeen, Aberdeen, UK; c Umeå Centre for Global Health Research, Department of Epidemiology and Global Health, Umeå University, Umeå, Sweden; d MRC/Wits Rural Public Health and Health Transitions Research Unit [Agincourt], School of Public Health, Faculty of Health Sciences, University of the Witwatersrand, Johannesburg, South Africa; e National Health Service, Grampian, UK; f Independent Public Health and Nutrition Consultant, Nelspruit, South Africa; g Institute for Global Health and Development, Queen Margaret University Edinburgh, Musselburgh, UK; h Department of Community Medicine, College of Medical Sciences, University of Calabar, Calabar, Nigeria; i Department of Global Health and Population, Harvard T. H. Chan School of Public Health, Harvard University, Boston, MA, USA; j Studies of Epidemiology of Epilepsy in Demographic Surveillance Systems (SEEDS) – INDEPTH Network, Accra, Ghana

**Keywords:** Non-communicable diseases, verbal autopsy, participatory research, civil registration and vital statistics, South Africa

## Abstract

**Background**: NCDs are non-infectious, long-term conditions that account for 40 million deaths per annum. 87% of premature NCD mortality occurs in low- and middle-income countries.

**Objective**: The aims were:develop methods to provide integrated biosocial accounts of NCD mortality; and explore the practical utility of extended mortality data for the primary health care system.

**Methods**: We drew on data from research programmes in the study area. Data were analysed in three steps: [a]analysis of levels, causes and circumstances of NCD mortality [n = 4,166] from routine census updates including Verbal Autopsy and of qualitative data on lived experiences of NCDs in rural villages from participatory research; [b] identifying areas of convergence and divergence between the analyses; and [c]exploration of the practical relevance of the data drawing on engagements with health systems stakeholders.

**Results**: NCDs constituted a significant proportion of mortality in this setting [36%]. VA data revealed multiple barriers to access in end-of-life care. Many deaths were attributed to problems with resources and health systems [21%;19% respectively]. The qualitative research provided rich complementary detail on the processes through which risk originates, accumulates and is expressed in access to end-of-life care, related to chronic poverty and perceptions of poor quality care in clinics. The exploration of practical relevance revealed chronic under-funding for NCD services, and an acute need for robust, timely data on the NCD burden.

**Conclusions**: VA data allowed a significant burden of NCD mortality to be quantified and revealed barriers to access at and around the time of death. Qualitative research contextualised these barriers, providing explanations of how and why they exist and persist. Health systems analysis revealed shortages of resources allocated to NCDs and a need for robust research to provide locally relevant evidence to organise and deliver care. Pragmatic interdisciplinary and mixed method analysis provides relevant renditions of complex problems to inform more effective responses.

## Background

In recent years, pronounced shifts have occurred in patterns of communicable to non-communicable diseases (NCDs). Globally, NCDs account for around 40 million deaths per annum, having increased by seven million since 2008 [1]. 87% of premature mortality and morbidity is borne in low- and middle-income countries (LMICs) and 40% is preventable [1]. These shifts have been linked to increasingly effective infectious disease control, decreasing fertility and increased life expectancy [2–5]. In high-income countries [HICs], this transition occurred in the early twentieth century. In LMICs, rapid urbanisation, industrialisation and related lifestyle changes have led to increasing burdens of NCDs combining with ‘unfinished agendas’ of infectious diseases, malnutrition, maternal and child mortality. NCDs, therefore, seriously challenge and compromise weak and under funded health systems in resource constrained settings [2,3,6].

South Africa is a country with a diversified economy undergoing a dynamic and protracted epidemiological transition. From the 1990s to 2004, HIV/AIDS devastated the country and communicable disease trajectories reversed [3]. While significant improvements were observed after2007, communicable diseases have not returned to pre-1990s levels [3]. South Africa is also one of the most unequal societies in the world [7], with avoidable death and disability deeply patterned by historical legacies of colonialism and apartheid and economic inequalities post-apartheid [7]. The result is a high and highly unequal distribution of illness. HIV-related mortality in the black population, for example, is 50 times that of whites [8,9]. In this context, NCDs form part of a ‘quadruple burden’ of avoidable mortality in South Africa with communicable diseases, maternal and child mortality, and mortality owing to violence, within which NCDs are predicted to be the leading cause of death by 2030 [2,7].

In South Africa and particularly in rural locations, reliable routine information on burden of disease is critical to inform policy and planning. At national level, Civil Registration and Vital Statistics (CRVS) systems record information on births, cause of deaths, marriages and divorces, and compile and disseminate these data to inform governments on population demographics [10]. Despite the need for reliable information on the basic characteristics of people’s lives, there is a significant shortage of data on vital events due to underfunded and incomplete CRVS systems in many LMICs [10–12]. Until recently, 62% of deaths worldwide were thought to pass un-recorded in terms of medical cause[s] [12,13]. In 2015, 27 million of the 56 million deaths that occurred globally were estimated to have been recorded meaningfully [14]. While South Africa has generally high CRVS coverage due to the National Births and Deaths Registration Act of 1992 [15,16], assessments of global cause of death [COD] data have identified the quality of information to be low [11].

In lieu of functioning CRVS, Verbal Autopsy [VA] has emerged as a pragmatic alternative to the provision of routine mortality data where there is no or incomplete registration. VAs are questionnaire interviews conducted by trained fieldworkers with final caregivers of deceased individuals on their medical signs and symptoms before death, which are later analysed to determine probable medical causes [14]. When done in sufficient numbers, VA allows for levels and causes of death to be reported in otherwise unregistered populations. The method is widely established, having been used in over 45 LMICs for over 25 years [14]. As global deficits in vital data systems are increasingly acknowledged, the utility of VA for routine data systems has been widely advocated for at regional and international levels [10,17,18].

In this paper with a focus on NCD, we present work conducted to support the contextualisation and utilisation of routine VA data for district health systems as follows:

1. We present data from Inter-VA 5 indicators to routinely collect data on critical limiting circumstances at and around the time of death. These enhance data on biomedical cause [18,19], and have been adopted into international VA standards [19–21];
2. We employ participatory research on complex health problems, centralising insights from those for whom the issues are most directly relevant [22,23]. Engaging ‘communities as researchers’ promotes empowerment and inclusion in the processes of producing and using new knowledge [24–26];
3. We draw on health policy and systems research [HPSR] to improve the practical utility of research evidence in the health system considering how data and evidence can be more relevant to local contexts, needs and situations [27–29].

### Aims and objectives

The work was based on the premise that premature NCD mortality arises as a result of multiple factors of social, medical and behavioural origin. To effectively intervene, it is necessary to understand NCDs in these terms and use this understanding at the local level to inform incremental change, for example reducing preventable NCD deaths. The aims were therefore to: (a) develop broader biosocial understandings of NCD mortality combining health surveillance with local knowledge and; (b) consider the practical utility of these data in the district health system. The objectives were to: (a) combine VA surveillance data with qualitative data developed via participatory research; (b) explore explanatory gains from integrating survey data with community-developed evidence; and (c) determine the practical relevance of these data in the district health system.

## Methods

### Study setting and design

**Figure 1.**
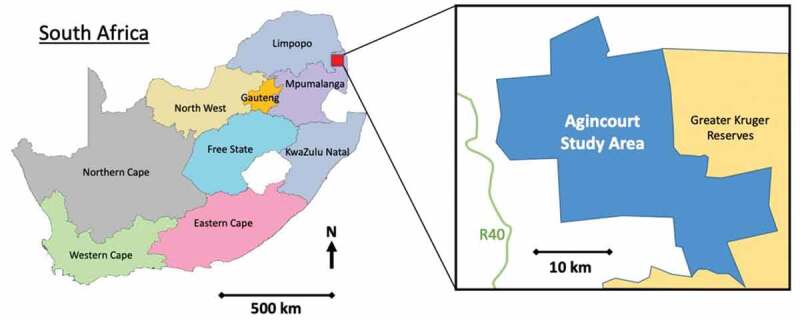
Map of South Africa, and Agincourt HDSS

We drew on data from the PHEVA (Public Health Evaluation and Verbal Autopsy) and VAPAR (Verbal Autopsy with Participatory Action Research) programmes [8,30,31] in the MRC/Wits Rural Public Health and Health Transitions Research Unit which operates the Agincourt Health and Socio-Demographic Surveillance System (HDSS) in rural northeast South Africa (Figure 1). The site covers 450 km^2^ containing 31 villages within the local Bushbuckridge municipality in Mpumalanga Province, close to the Mozambique border [32]. Established in 1992, Agincourt is one of the most developed HDSS sites in South Africa [32]. Data were analysed in three stages: (a) analysis of levels, causes and circumstances of NCD mortality from VA data in the Agincourt HDSS, and of qualitative data on lived experiences of NCDs in rural villages drawn from participatory research; (b) analytical integration, identifying areas of convergence and divergence between the quantitative and qualitative analyses; and (c) district health systems appraisal of new data and data systems.

(Hove J, D’Ambruoso L, Mabetha D, et al. ‘Water is life’: developing community participation for clean water in rural South Africa. *BMJ Global Health*2019;4:e001377. doi:10.1136/bmjgh-2018-001377.

### Data collection

VA data were obtained from the 2012 to 2016 census update rounds in the Agincourt HDSS. For all deaths identified, final caregivers were interviewed using a questionnaire based on the 2012 WHO VA standard [19], with levels and medical causes determined using InterVA-5.1 [17,19,33]. The PHEVA data included transcripts from focus group discussions (FGDs) comprised of 24 people, held in three villages in 2013, selected to vary by demographics and health service access, and in which discussion groups were convened to represent community-based service users and providers (2 groups), and women (1 group) [8]. A series of discussions were held on HIV/AIDS, violence, stroke and epilepsy as conditions reflecting the leading CODs in the site and around which pronounced social and cultural norms exist [34]. The qualitative element of this study is concerned with stroke and epilepsy. The results on HIV and violence and a fuller description of the data collection processes are provided elsewhere [8]. Finally, the practical relevance of the data was appraised with stakeholders from the Department of Health at local, district and provincial levels, which involved seven formal engagements with frontline service providers, programme managers and planners on research co-design and data interpretation, considering the practical utility of new data and data processes. This was supplemented by several informal exchanges and discussions during the course of both research programmes (Table 2).

### Patient and public involvement statement

The research was developed to better understand NCDs in rural South Africa. We did not work with patients directly but with people living in rural villages who were involved in study design in terms of nominating focus topics and expanding the participant base. This approach will continue to be followed in the evaluation activities, as it is a core component of our programme and evaluation approach.

### Data analysis

From the VA data, categorised according to the WHO standards for VA cause of death groups [21] (Appendix 1), we computed cause-specific mortality fractions (CSMFs) by age and sex. InterVA-5 attributes each death to likelihoods across up to three probable causes, with any residual component assigned as indeterminate. Hence, numbers of deaths assigned to each cause category are not necessarily integer values. Circumstances of Mortality Categories (COMCATS) were quantified in the following categories: Traditions, Emergencies, Health Systems, Inevitability, Recognition, Resources and deaths may be assigned to multiple combinations of these categories (Table 1). Further details on COMCATs using InterVA-5 are published elsewhere [35]. As the data were derived from complete population enumeration rather than on the basis of a sample, tests of significance were not relevant.

**Table 1.** Circumstances of mortality categories (COMCATs). COMCATs description

ComCAT	Description
Traditions	Traditional practices or beliefs influenced health seeking behaviour and the pathway to death Emergencies Sudden, urgent or unexpected conditions leading to death, which probably precluded life-saving actions
Recognition	Lack of recognition or awareness of serious disease (e.g. symptoms or severity) negatively influenced health seeking behaviour
Resources	Inability to mobilise and use resources (e.g. material, transport, financial) hindered access to health care
Health Systems	Problems in getting health care despite accessing health facilities (e.g. related to admissions, treatments and medications)
Inevitability	Death occurred in circumstances that could not reasonably have been averted (e.g. very elderly or recognised terminal conditions)
Multiple	A combination of the above categories affected the pathways to death; no single factor predominated

For the narrative data, thematic analysis was performed to develop a descriptive overview via a combined inductive/deductive framework analysis. The process involved familiarisation, and identification of patterns and themes, which were used to develop codes to (re)-apply to the data iteratively until a reasonable point of saturation occurred [36]. NVivo version 10 [37] was used for data management and coding with periodic checking and verifying. In order to consider the practical relevence of the data, we analysed presentations, registers, minutes, observational notes and reflective journals to develop accounts of the perspectives of health systems stakeholders on the evidence they have access to and require, and how information can inform policy, planning and management. This was also based on a combined inductive/deductive approach, whereby latent and manifest meaning were derived from the data to develop accounts of the district health systems utility of new data and data processes [36].

### Ethical considerations

Informed consent was received from all participants. Routine VA surveys in the Agincourt HDSS are approved by the Human Research Ethics Committee (HREC) (Medical) of the University of the Witwatersrand, and informed consent is obtained at the beginning of surveillance and reaffirmed regularly at individual, household and community levels. The participatory research (PHEVA) and health systems engagement protocols were reviewed and approved by Wits HREC (Medical) and the University of Aberdeen College Ethics Review Board (CERB) (clearances: M121039, M1704115, M171050, M171050 amendment, CERB/2017/4/1457; CERB/2017/9/1518; CERB/2017/9/1518). Permission was secured from Mpumalanga provincial health authority (MP_201712_003).

## Results

### Medical causes and circumstances of death

Verbal autopsy interviews were undertaken for 4,111 deaths from 2012 to 2016, and causes were successfully assigned to 84% of deaths, with 16% remaining of indeterminate cause. NCDs contributed a significant percentage, 38%, of the total mortality burden and predominantly affected people over 65 years, to which 51% of NCD deaths were assigned, followed by 50–64 years (21%) and 15–49 years (26%). Only 2% of NCD deaths occurred in children under 15 years. Infectious conditions accounted for 34% of all deaths, with HIV/AIDS-related mortality (13%) and pneumonia (11%) the most common causes of infectious death (Table 3).

**Table 2.** Summary of different data components

	Methods	Source	Data types
Verbal Autopsy data	Inter-Va 5	2012–2016 Agincourt census	Quantitative, verbal autopsy cause of death records
Circumstance of mortality data	COMCAT	2012–2016 Agincourt census	Quantitative, circumstance of mortality data
Community discussion on NCDs	CBPR	PHEVA Study	Qualitative, transcripts from focus group discussions on Stroke and Epilespy.
Health system appraisal	PAR	VAPAR study	Qualitataive, knowledge exchange via secondary reports, meeting summaries and resources.

**Table 3.** Cause of death groups by age and sex sub-groups, number of deaths (column %)

Age and sex groups	< 28 days	1–11 months	1–4 years	5–14 years	15–49 years	50–64 years	> 65 years	Female	Male	Total
**Cause of death groups**										
Non-communicable	-	5.3 (4.4)	14.2 (10.4)	12.6 (12.5)	412.6 (24.4)	329.4 (40.1)	820.2 (61.5)	857.2 (42.2)	737.1 (35.4)	1594.2 (38.8)
Infectious	-	84.2 (70.2)	79.9 (58.9)	45.3 (44.9)	689.5 (40.7)	213.8 (31.4)	283.6 (21.3)	672.7 (33.1)	723.5 (34.7)	1396.2 (34.0)
External	-	2.0 (1.7)	12.2 (9.0)	19.8 (19.6)	260.8 (15.4)	31.7 (4.7)	20.9 (1.6	71.8 (3.5)	275.5 (13.2)	347.2 (8.4)
Neonatal	23.9 (56.9)	1.0 (0.8)		-	-	-	-	9.6 (0.4)	15.3 (0.7)	24.9 (0.6)
Maternal	-	-	-	0.7 (0.7)	80.7 (4.8)	-	-	81.3 (4.0)	-	81.3 (2.0)
Indeterminate	18.1 (43.1)	27.6 (23.0)	29.8 (21.9)	22.6 (22.4)	250.5 (14.8)	105.1 (15.5)	209.4 (15.7)	340.5 (16.8)	326.6 (15.7)	667.1 (16.2)
Female	21 (50.0)	63 (53.0)	51 (37.5)	35 (34.7)	804 (47.5)	281 (41.3)	776 (58.2)			2033 (49.5)
Male	21 (50.0)	57 (47.0)	85 (62.5)	66 (65.3)	890 (52.5)	399 (58.7	558 (41.8)			2078 (50.5)
Total	42 (100.0)	120 (100.0)	136 (100.0)	101 (100.0)	1694 (100.0)	680 (100.0)	1334 (100.0)	2031 (100.0)	2085 (100.0)	4111 (100.0)

Male and female percentages of deaths due to NCDs were 35% versus 42% (Table 3). There were few differences in NCD causes between males and females. Cancer was a key feature of the NCD mortality. Neoplasms in six WHO VA cause categories accounted for 38% of NCD deaths, and 12% of all deaths. Cancer accounted for a slightly higher percentage of male NCD deaths (36%) versus deaths in females (27%). Cardiac conditions accounted for 27% of NCD deaths, epilepsy and stroke accounted for 1% and 11% respectively.

In terms of COMCATs, issues of recognising problems in terms of severity of illness in the days preceding death were associated with deaths most frequently (22%)). This was closely followed by problems with mobilising resources to seek and receive care in the days preceding death (21%) and within the health system (19%). Inevitability was associated with 14% of deaths. The percentage of deaths associated with emergencies was 17%. The traditions domain, such as the use of traditional medicine at or around the time of death, was the lowest scoring COMCAT with only 3% of deaths attributed to this category. Of all the deaths, 4% were determined to have been associated with multiple circumstantial categories (Tables 4 and 5).

**Table 4.** Numbers of deaths by circumstances of mortality category and cause of death group (column %)

Circumstances of mortality Category	NCDs	Infectious	External	Neonatal	Maternal	Indeterminate	Total
Traditions	49.1 (3.1)	49.4 (3.5)	1.3 (0.4)	2.0 (8.0)	4.3 (5.3)	24.0 (3.6)	130 (3.2)
Emergencies	140.6 (8.8)	63.9 (4.9)	297.1 (85.6)	3.0 (12.0)	35.0 (43.1)	145.8 (21.9)	685 (16.7)
Health Systems	288.7 (18.1)	363.1 (45.8)	1.4 (0.4)	6.7 (26.9)	17.8 (21.9)	76.4 (11.5)	754 (18.3)
Inevitability	365.4 (22.9)	82.1 (5.9)	18.0 (5.2)	3.0 (12.0)	2.5 (3.1)	104.1 (15.6)	576 (14.0)
Recognition	394.8 (24.8)	408.5 (29.3)	-	0.9 (3.6)	0.9 (1.1)	72.9 (10.9)	878 (21.3)
Resources	309.4 (19.4)	371.5 (26.6)	25.8 (7.4)	0.8 (3.2)	17.1 (21.0)	117.4 (17.6)	842 (20.5)
Multiple Categories	46.4 (2.9)	58.0 (4.2)	2.7 (0.8)	8.4 (33.7)	3.8 (4.7)	126.6 (19.0)	246 (6.0)
TOTAL	1594.2 (100)	1396.2 (100)	347.2 (100)	24.9 (100)	81.3 (100)	667.1 (100)	4111 (100)

In the NCD deaths, the COMCATs largely reflected the overall pattern, with limits on resources and health systems problems being reported in 19% and 18% of cases respectively. However, there was a difference in COMCATs between total and NCD deaths in the inevitable category, at 23% for NCD deaths as opposed to 9% for non-NCD deaths; this may be explained by the nature of NCD deaths often being anticipated by the time they occur. Conversely, the emergencies category applied to 9% of NCD deaths, but to 22% of non-NCD deaths (Table 4).

### Local knowledge

Corroborating the main VA findings, in the participatory research, access to care for stroke and epilepsy was repeatedly described as limited, with poverty a recurring theme. Poverty was described as directly impacting people’s abilities to pay for travel to clinics and pharmacies, and to obtain medication. Transport to clinics, clinic-opening hours and long waiting times in clinics were also described as common barriers to access.

‘ … she can’t go [to the clinic] by herself and has no one to fetch the medication … every time she goes she needs to hire a private car’ [Village 2; Community official; Discussion on Stroke]

Care-seeking decisions were also influenced by perceptions of low quality of care in clinics and widespread mistrust in clinic staff. Participants repeatedly recounted problems with interpersonal aspects of care, particularly around lack of dignity, respect and confidentiality. Participants reported preferring to visit clinics or facilities in neighbouring villages to maintain privacy and confidentiality, expressing willingness to die at home rather than visit clinics where dignity and respectful care were not always upheld. The importance of confidentiality was acknowledged in the village-based discussion groups.

“ … once they (disclose confidential information) I will never go back again because it’s dangerous … I will try by all means to go to [location A] or [B] to get treatment.” [Village 1; Religious leader]

“People decide to die at home because of this (confidentiality breaches in clinics)” [Village 2; community official]

“ … you won’t hear me disclosing people’s illnesses … It’s between me and them” [Village 3; Traditional healer]

The narratives revealed traditional beliefs as further influences on care seeking. *‘Xifulana’* was referred to on many occasions as curses and bewitchment that caused stroke. Many participants recounted that the first point of help for *Xifulana* should be a traditional healer. Others described not knowing whether to go to a healer or to hospital in an acute situation. Although it was acknowledged that seeking traditional treatment could result in conditions progressing to a fatal degree, hospital treatment for *Xifulana* was also perceived to be harmful.

“ … we are unable to differentiate stroke and Xifulana, (when we) realise that she has *not* been bewitched [it is] too late” [Village 1; Religious leader; Discussion on Stroke]

“ … when someone is attacked by a stroke … If he collapses and falls down we will start shouting … *witch, witch, witch*” [Village 1; Religious leader Discussion on Stroke]

“ … (if) you have been bewitched … they need to take you to a traditional healer … in hospital they may inject you and make it worse.” [Village 2; Family Member; Discussion on Stroke]

Traditional beliefs around epilepsy were also described, often with reference to *‘Ringhadi’*, the presence of a snake in the abdomen. Many participants viewed traditional medicine as essential for the treatment of *Ringhadi*, and that medical treatment could only stabilise the condition. Participants reported that babies should receive traditional remedies preventatively and generally described epilepsy as a ‘respected illness’, one that was not discussed and perceived as dangerous to talk about. It was also understood that epilepsy could be both inherited and transmitted.

“Ringhadi … this epilepsy thing is a snake with two heads inside the stomach … and if you don’t give herbs … the snake develops another third head … if you use traditional herbs in the early stages … it won’t happen” [Village 3; Traditional healer; Discussion on Epilepsy]

“ … to say he is healed, the snake must die and come out. Hospital medication doesn’t do such things … they just manage to control the situation but not completely heal it” [Village 3; Family member; Discussion on Epilepsy]

“ … if you talk about epilepsy in the presence of a child it might happen that he got infected by it” [Village 2; Women of reproductive age; Discussion on Epilepsy].

Although widespread use of traditional medicine was apparent, some traditional treatments were described as harmful, with severe consequences in some instances. Participants detailed a treatment called ‘Steaming’ used by traditional healers in the treatment of stroke. During this treatment, a room was heated up using fire and steam and individuals suspected of having a stroke are left in the room covered in blankets.

“They will cover you with the blankets and it is terrible, you can even die there [at traditional practices] … you can even suffocate (during steaming)” [Village 1; community health volunteer; Discussion on Stroke]

Participants shared detailed knowledge about signs and symptoms of stroke and epilepsy, as well as on a range of causative agents and risk factors of differing severity. Symptoms were reported including: loss of function and sensation, paralysis and memory loss for stroke; and uncontrollable movements, fainting, frothing at the mouth and incontinence for epilepsy. Participants noted that seizures happen suddenly and without warning and that, individuals can be a danger to themselves.

Similarly, risk factors for stroke were identified as high salt intake, high blood pressure, low physical activity and poor diet. Stress, described as ‘thinking too much’, was a repeated and persistent theme in the discussions on risk factors and root causes for both conditions. Stress as a result of poverty was repeatedly recounted as a significant factor contributing to the exacerbation of issues such high blood pressure and as poor diet, which were in turn, thought to contribute towards the risk of stroke. Poverty was identified as a root cause of both conditions.

“Poverty is dangerous … they went to bed without food, they have to think twice … she end up falling down and attacked by stroke … It can be stress that attacked her and she had a stroke … poverty is the main issue” [Village 1; Religious leader;; Discussion on Stroke]

### Integrating interpretations

The VA data revealed the extent of difficulty mobilising resources for care [direct and indirect costs] in the days before death in all deaths and for NCD deaths. The narrative data provided rich detail on the specific mechanisms through which lack of resources limits access to care in the acute care seeking period in the final days before death – ‘*every time she goes to the clinic she needs to hire a private car*’ – and exposes individuals to risk – ‘*they went to bed without food, they have to think twice … she end up falling down and attacked by stroke*’. As stated by a participant ‘*poverty is the main issue*’. This lack or resources may manifest at and around the time of death in not calling for help and not travelling to facilities.

The qualitative data also revealed the extent of perceptions of poor quality care in local clinics as a critical issue affecting care seeking. Participants provided powerful descriptions of preferring to die at home as opposed to travelling to facilities, where they perceived they would be treated poorly. These findings further contextualise the ‘health system’ COMCAT that was reported as problematic factor for 18.3% of total deaths. This is further supported by the wider literature, which identifies pervasive issues with quality of care in this setting.

Problems related to use of traditional therapies at and around time of death for all deaths were not identified as a major problem in the VA data, while the narratives described pervasive use of traditional medicine and widely held traditional beliefs. In terms of the latter, stroke and epilepsy were considered in both traditional and biomedical terms, with negative consequences for care seeking and treatment.

The relatively low reporting of preference for traditional therapies as critical limiting circumstantial factor in VA for all deaths and NCD deaths may not represent lack of use of traditional medicine but rather the timeframe of its use. For example, individuals may have used traditional medicine at earlier stages of the disease opposed to around the time of death. The circumstances categories ask questions about events at or around the time of death, whereas the FGDs considered conditions in general.

### Health systems appraisal

The PHEVA and VAPAR methods were appraised over a series of formal and informal engagements with programme managers and planners in the local, district and provincial health system in 2016–18, during the dissemination of results and consultation on future research and service development. In these interactions, we gained further insights into the acceptability and utility of the data for use in the health system.

Department of Health colleagues at different levels [province, district and local] consistently recounted that the health system has little routine data that is reasonably accurate. Health systems stakeholders noted that data on deaths outside facilities, and on the critical limiting circumstances and events of those deaths, were of critical relevance to inform service organisation and delivery. It was seen as important that VA data from the Agincourt HDSS are reasonably generalisable to the district. District stakeholders also frequently emphasised the importance of statistics being accessible to lay persons. Health planners and managers reported finding the qualitative participatory research data powerful and more suitable to convey human stories of exclusion from access to health systems. The combination of the statistical and qualitative data was found to be useful in interpreting the data.

Engaging in the health system in different levels and sections also revealed that NCDs are chronically under-funded at province and district level]. Considering the scale of NCD mortality is commensurate with that of infectious diseases, which receive a substantially larger proportion of health funding from government and a range of national and international agencies, it was a finding of concern that the NCD directorate is severely under-resourced in terms of budget and personnel allocation, as well as lacking in robust and timely routine data on the magnitude of the problem.

Finally, through several engagements of increasing depth in the district and provincial health system, we were able to develop an understanding of the extent of the well-established policy-implementation gap]. There were many accounts of large numbers of vertical policies and strategies that take little account of the operational contexts in which they are implemented. As such, when implemented, there are chronic problems and the system is often overwhelmed with large numbers of policies, programmes and strategies]. In this sense, the data were seen to have the potential to reflect the reality of policy implementation and provide a relevant input to centralised policy and strategy.

## Discussion

Our aims were to develop biosocial accounts of NCD mortality in a rural area in South Africa and consider the practical utility of the data and evidence in the rural primary health care system. The VA data revealed a double burden of NCDs and infectious diseases, reflecting a dynamic and protracted epidemiological transition in South Africa [2,3,5]. NCD mortality can be attributed to a rural population undergoing significant demographic and social changes inclusive of ageing, changes in lifestyle, diet and activity [38]. This epidemiological transition may also contribute to the high percentage of NCD deaths included in the ‘inevitable’ COMCAT, as these are typically long-term chronic conditions that are often seen in the elderly. However, these are also conditions that that in later stages of the disease are harder to treat successfully, therefore those not diagnosed and treated in a timely manner often have poorer outcomes and places undue burden on rural PHC.

The COMCAT data revealed multiple problems with resources to access care in the final days before death. Over a fifth of deaths in both the total deaths and NCD category were attributed to problems associated with mobilising resources for care in the acute period before death, which materialised in not calling for help and not travelling to a hospital in the days before death. Despite free primary healthcare in South Africa, the chronic nature of poverty and NCDs is likely to lead to unaffordable care overall in both direct and indirect costs for patients and families, and with individuals less likely to call for help or travel to a facility prior to the time of death [36]. These findings underscore the importance of continuous, robust health and socio-demographic surveillance to inform more integrated policy, planning and service delivery [2,39].

The participatory research contextualised commonly experienced barriers to access, providing detailed explanations of how and why they exist. Community knowledge on stroke and epilepsy revealed how perceptions of poor quality of care, especially related to inter-personal elements around confidentiality, act as major deterrents to visiting facilities. These findings are consistent with research on poor quality of integrated management of chronic diseases in the sub-district, including lack of resources, confidentiality breaches and long waiting times in clinics in this setting [39,40]. Perceptions of health care quality have strong influences on care seeking, suggesting the need to consider quality and access as inextricably linked [41,42]. Indeed, composite indicators of country-specific quality and access have been proposed as a means to identify local priorities and monitor progress [43][44][45].

While participants had detailed knowledge of signs, symptoms and the need for timely treatment, illnesses were considered in both biomedical and traditional terms. This was particularly apparent for stroke and significantly influenced the type of care sought. There is evidence that for conditions with aetiologies that are not obvious, such as NCDs, individuals are more likely to assume traditional causes [34,46]. In South Africa, medical treatment is often secondary to that of traditional therapies, if considered at all, partly due to ease of access, affordability and acceptability [34,47]. For end-of-life care, this poses a particular issue with conditions likely to be in more advanced stages after exhausting traditional therapies [34]. The research reflects the need to embrace the complexity of non-communicable burdens of disease, the epidemiological transition that accompanies this, as well as the social realities of health and illness for people who deal with these issues. Perhaps, given the outlook that use of traditional therapies will continue as long as they are available, it is in the health system’s best interest to integrate and facilitate knowledge sharing between the two practices; South Africa has made policy moves in this direction [47,48].

### Health systems implications

Engaging with stakeholders in different levels and sections of the health system revealed the lack of robust, timely data on the extent and nature of the burden of NCDs, as well as serious shortages in human resource and budgetary allocations to NCDs at provincial level. This may be exacerbated by the fact that South Africa currently has no medium term strategic plan in place for NCDs and NCD plans are rarely implemented at a national level as responsibility for these fall to individual provinces and districts [49]. The lack of necessary planning and implementation highlights an apparent lack of prioritisation for NCDs, despite the high and increasing disease burden. The data and methods presented in this paper offer new approaches to mobilise and advocate for changes in the way in which NCDs are understood and addressed.

Given the low levels of resources allocated to NCDs in South Africa, developing models that leverage existing resources for infectious diseases to target NCDs may be necessary. An example of this is the Integrated Chronic Disease Management Model (ICDM), which aims to work with existing HIV programmes to scale up and support NCD care [39,50–53]. ICDM has been piloted in three provinces in South Africa, including Mpumalanga. Evidence from other locations suggest that the integrated HIV and NCD programmes enhance patient satisfaction and improve patient outcomes often due to increased implementation [39,54].

More broadly, the South African health system is deeply divided and the distribution of health benefits are distinctly pro-rich [7]. This is despite constitutional commitments to health as a human right, legislative shifts towards integrated care and Universal Health Coverage (UHC) and services without financial burden [55]. The government is currently in the process of implementing National Health Insurance (NHI) to provide an accessible, affordable and quality health service [55]. The reduction of financial burdens through NHI is important to address the multiple and reinforcing barriers to access and how perceptions of quality influence access. This study highlights that access is complex, and the ability to mobilise resources for care at and around the time of death is key and influenced by a range of social factors, processes and conditions. Improving affordability in direct costs may not resolve all issues, particularly those of indirect costs, such as travelling to and time spent at facilities, as well as interpersonal aspects of care when services are utilised. These elements must be considered, as well as the deeper social and structural determinants that underpin avoidable NCD mortality and the need to prioritise NCDs within the budget processes [54,55].

The implementation of NHI involves a comprehensive primary health care (PHC) re-engineering programme, which consists of connecting communities to services through PHC ward-based outreach teams that centralise community health workers’ (CHWs) roles in the community [55]. The mobilisation of communities through the expansion of CHWs will be invaluable to individuals with NCDs, particularly in rural settings. Such improvements in access may also serve to improve the relationships between people and the health system, through an improvement in connections and exchanges of information, addressing perceptions of poor quality of care and the implications for care seeking they impose.

However, interdisciplinary data from this study provides evidence of the complex burden of NCDs, and indicates the need for consistent, robust evidence providing meaningful evidence to inform the organisation and delivery of care. The pragmatic interdisciplinary and intersectoral analysis provides relevant renditions of the complexity of NCDs in rural South Africa, thereby providing greater insight in order to form more effective remedial responses.

Our recommendations are summarised in Table 6.

**Table 5.** Numbers of deaths by circumstances of mortality category and age group (column %)

Circumstances of mortality Category	< 28 days	1–11 months	1–4 years	5–14 years	15–49 years	50–64 years	65+ years	Total
Traditions	1 (2.4)	5 (4.2)	7 (5.1)	9 (8.9)	51 (3.0)	16 (2.4)	41 (3.1)	130 (3.2)
Emergencies	4 (9.5)	35 (29.2)	40 (29.4)	29 (28.7)	392 (23.1)	101 (14.9)	84 (6.3)	685 (16.7)
Health Systems	7 (16.7)	19 (15.8)	20 (14.7)	11 (10.9)	384 (22.7)	113 (16.6)	200 (15.0)	754 (18.3)
Inevitability	4 (9.5)	1 (0.8)	1 (0.7)	1 (1.0)	46 (2.7)	90 (13.2)	433 (32.5)	576 (14.0)
Recognition	1 (2.4)	36 (30.0)	52 (38.2)	29 (28.7)	295 (17.4)	160 (23.5)	305 (22.9)	878 (21.3)
Resources	1 (2.4)	13 (10.8)	10 (7.4)	14 (13.9)	441 (26.0)	155 (22.8)	208 (15.6)	842 (20.5)
Multiple Categories	24 (57.1)	11 (9.2)	6 (4.4)	8 (7.9)	85 (5.0)	45 (6.6)	63 (4.7)	246 (6.0)
TOTAL	42 (100)	120 (100)	136 (100)	101 (100)	1694 (100)	680 (100)	1334 (100)	4111 (100)

**Table 6.** Overall findings and recommendations

Findings	Recommendations
Premature and rapidly increasing mortality owing to NCDs is a critical health systems challenge. Understanding NCDs in rural South Africa is particularly crucial, considering the differences in health care and social support across societies.	VA data allowed a significant NCD burden to be quantified and revealed common barriers to access at and around the time of death. Highlighting the necessity of conducting research on NCDs in this location.
The reduction of financial burdens through NHI is important to address the multiple and reinforcing barriers to access and how perceptions of quality influence acces. These elements must be considered, as well as the deeper social and structural determinants that underpin avoidable NCD mortality and the need to prioritise NCDs within the budget processes.	The health systems analysis revealed a serious shortage of resource allocated to NCDs at provincial level and a need for consistent, robust approaches providing meaningful evidence upon which to organise and deliver care.
This study highlights that access is complex. These elements must be considered, as well as the deeper social and structural determinants that underpin avoidable NCD mortality and the need to prioritise NCDs within the budget processes.	The findings imply the value of conducting interdisciplinary research when considering complex problems, such as NCDs, and highlights the added value of pragmatic and plural analytical frameworks for more complete renditions of complex problems.
Interdiscplinary data from this study provides evidence of the complex burden of NCDs, and indicates the need for consistent, robust evidence providing meaningful evidence to inform the organisation and delivery of care.	The findings further imply the necessity that data is framed in a manner that is relevant, available and accessible to decision-makers, planners and managers in order to inform remedial responses.

### Methodological reflections

Several strengths and limitations are relevant to the findings and interpretations. The data were collected in a rural HDSS site. For the VA data, this allowed complete enumeration and so statistical tests of significance were not required. Although generalisability is often a concern with data obtained in HDSS systems, results from the Agincourt HDSS are generalisable with reasonable accuracy to the district, and are comparable with other villages nationally and regionally [57]. The results indicated a large proportion of deaths of indeterminate cause. As Inter-VA methods evolve, this proportion may narrow.

Furthermore, given that the circumstances of mortality categories vary between different causes of death [18], these categories can potentially inform the processes of establishing medical causes. The circumstances of the indeterminate deaths display similar characteristics to acute cause of death categories and were predominately among adult males.

A further consideration is the time period related to the VA and participatory research. The VA data is concerned with signs, symptoms and events at and around time of death, whereas the FGDs considered conditions in more general terms. It is reasonable therefore to expect a degree of difference in the perspectives gained from the different methods. Considering the data sets as complementary rather than directly comparable allowed explanatory gains to be achieved, particularly related to describing and explaining resources and care within the health system. These provided insights into the experience of NCDs in this setting, and specifically in terms of the ways in which these impact care-seeking processes.

## Conclusions

Premature and rapidly increasing mortality owing to NCDs is a critical health systems challenge. Understanding NCDs in rural South Africa is particularly crucial, considering the differences in health care and social support across societies. VA data allowed the NCD burden to be quantified and revealed pervasive problems with resources, which could potentially deter people from seeking help and travelling to health facilities at and around the time of death. Participatory research contextualised these barriers, providing rich explanations of why they exist. There were reports of chronic poverty in rural villages constraining people’s abilities to mobilise resources to seek and receive good quality care in acute situations, as well as predisposing populations to risks more generally. Strong views about low quality of care in clinics acted to further constrain access. Despite detailed understanding of signs, symptoms and severity, disease aetiologies were also conceived of in traditional terms of curses and witchcraft, which had further negative influences on seeking care at and around the time of death. The health system analysis identified the need for consistent, robust approaches providing meaningful evidence with which to strengthen the health system’s capacity to provide NCD services, including adequate resourcing, specifically through implementation of the NHI and PHC re-engineering. This study highlights the benefit of pragmatic and plural analytical frameworks. It demonstrates how they provide more complete renditions of complex problems that are relevant, available and accessible to decision-makers, planners and managers in order to inform more effective remedial responses, demonstrating the value of interdisciplinary in order to generate evidence on complex problems, such as NCDs.

## Paper context

Deaths due to non-communicable diseases are increasing rapidly in South Africa. They are predicated to be the leading cause of death by 2030. There is a significant shortage of data on vital events due to underfunded and incomplete CRVS systems. This study highlights the added value of pragmatic and plural analytical frameworks for more complete renditions of complex problems. The findings imply the value of conducting interdisciplinary research when considering complex problems, such as NCDs;Department for International Development (DFID)/Medical Research Council (MRC)/Wellcome Trust/Economic and Social Research Council (ESRC) [(MR/N005597/1 and MR/P014844/1).]

## Appendix 1.

**Appendix 1.** Causes of death and cause of death categories

Infectious	Acute respiratory infection incl. pneumonia Diarrhoeal diseases Haemorrhagic fever HIV/AIDS related death Malaria Meningitis and encephalitis Other and unspecified infect disease Pulmonary tuberculosis Sepsis (non-obstetric)
NCD	Acute abdomen Acute cardiac disease Asthma Breast neoplasms Chronic obstructive pulmonary disease Diabetes mellitus Digestive neoplasms Epilepsy Liver cirrhosis Oral neoplasms Other and unspecified cardiac disease Other and unspecified NCD Other and unspecified neoplasms Renal failure Reproductive neoplasms male/female Respiratory neoplasms Severe anaemia Severe malnutrition Stroke
External	Road traffic accident Other transport accident Accidental fall Accidental drowning and submersion Accidental expos to smoke fire & flame Contact with venomous plant/animal Acid poisoning & noxious subs Intentional self-harm Assault Exposure to force of nature Other and unspecified external CoD
Neonatal	Birth asphyxia Congenital malformation Neonatal pneumonia Neonatal sepsis Prematurity Other and unspecified neonatal CoD
Maternal	Abortion-related death Obstetric haemorrhage Pregnancy-induced hypertension Pregnancy-related sepsis Ectopic pregnancy Anaemia of pregnancy Other and unspecified maternal CoD
Indeterminate	NULL
